# Role of Capsule and O Antigen in the Virulence of Uropathogenic *Escherichia coli*

**DOI:** 10.1371/journal.pone.0094786

**Published:** 2014-04-10

**Authors:** Sohinee Sarkar, Glen C. Ulett, Makrina Totsika, Minh-Duy Phan, Mark A. Schembri

**Affiliations:** 1 Australian Infectious Disease Research Centre, School of Chemistry and Molecular Biosciences, The University of Queensland, Brisbane, Queensland, Australia; 2 School of Medical Sciences, Griffith Health Institute, Griffith University, Gold Coast, Queensland, Australia; Université d'Auvergne Clermont 1, France

## Abstract

Urinary tract infection (UTI) is one of the most common bacterial infections in humans, with uropathogenic *Escherichia coli* (UPEC) the leading causative organism. UPEC has a number of virulence factors that enable it to overcome host defenses within the urinary tract and establish infection. The O antigen and the capsular polysaccharide are two such factors that provide a survival advantage to UPEC. Here we describe the application of the *rpsL* counter selection system to construct capsule (*kpsD*) and O antigen (*waaL*) mutants and complemented derivatives of three reference UPEC strains: CFT073 (O6:K2:H1), RS218 (O18:K1:H7) and 1177 (O1:K1:H7). We observed that while the O1, O6 and O18 antigens were required for survival in human serum, the role of the capsule was less clear and linked to O antigen type. In contrast, both the K1 and K2 capsular antigens provided a survival advantage to UPEC in whole blood. In the mouse urinary tract, mutation of the O6 antigen significantly attenuated CFT073 bladder colonization. Overall, this study contrasts the role of capsule and O antigen in three common UPEC serotypes using defined mutant and complemented strains. The combined mutagenesis-complementation strategy can be applied to study other virulence factors with complex functions both *in vitro* and *in vivo*.

## Introduction

Urinary tract infections (UTIs) are one of the most common types of bacterial infection in humans. Approximately 150 million cases of UTI are estimated to occur across the globe annually [1]. UTI occurs frequently in women; it is estimated that 40–50% of adult healthy women experience at least one UTI episode in their lifetime [2]. Catheterized patients, the elderly and children are also at a higher risk for UTI [3]–[5]. If untreated, UTI may progress from the bladder to the kidneys and even cause life-threatening urosepsis. The estimated annual cost of treating UTI in the USA alone is greater than $1.6 billion, thus emphasizing the global economic burden these infections place on health care systems [2].

Uropathogenic *Escherichia coli* (UPEC) is the primary causative organism in more than 80% of all UTI cases [6]. UPEC isolates exhibit a high degree of genetic diversity, primarily due to the possession of specialized virulence genes located on mobile genetic elements called pathogenicity islands [7], [8]. Although no single virulence factor is uniquely definitive of UPEC, their ability to cause symptomatic UTI is enhanced by adhesins (e.g. type 1, and P fimbriae), toxins (e.g. hemolysin), iron acquisition systems and cell surface polysaccharides [9]–[11]. The capsule and the O antigen represent two cell-surface associated polysaccharides that contribute to UPEC virulence [12]–[14]. These extracellular polysaccharides protect UPEC against phagocytosis by immune cells such as neutrophils and inhibit complement-mediated killing [15], [16].

Bacterial lipopolysaccharide (LPS) plays a primary role in mediating UPEC resistance to the bactericidal activity of human serum [17]. LPS consists of the highly conserved lipid A-core and repeating O antigen subunits that differ greatly between strains based on the sugar residues and their linkage patterns within the repeating subunits [18]. More than 180 different serotypes have been reported for *E. coli* [18], [19], with some O antigen types such as O1, O2, O4, O6, O7, O8, O16, O18, O25, and O75 being common among UPEC strains [20]. This variation is imparted by differences in the genes that encode for glycosyltransferase enzymes that modify O antigen structure. The lipid A-core and the O antigen subunits are assembled in separate pathways that come together for ligation at the inner membrane [21]. WaaL is a conserved O antigen ligase and the only known membrane enzyme implicated in the ligation of pre-assembled undecaprenyl-diphosphate (Und-PP)-linked O antigen to lipid A-core oligosaccharide [22]. *E. coli* K-12 *waaL* deletion mutants are unable to cap the lipid A-core and are therefore disrupted in the expression of O antigen at the cell surface [23].

Like the O antigen, there is also significant variation in the *E. coli* capsule. More than 80 distinct types of *E. coli* capsule have been identified, with four major groups defined based on genetic assembly, biochemistry and physical attributes [24]. The group 2 capsular polysaccharides have mostly been associated with UPEC strains [12], [25]. The role of the capsule in protecting UPEC from phagocytosis has been defined [26], [27], however, its role in resistance to serum-mediated killing is unclear, with several conflicting reports in the literature [28]–[31]. Understanding the role of the capsule in UPEC serum resistance is further complicated by the use of multiple strains with different O antigen and capsule types. For example, a multi-strain study by Stawski *et al.* found that the contribution of capsule to serum resistance varied between strains and ranged from being important to having no protective role [32]. The genes involved in capsule production are located in the *kpsFEDUCS* operon [24], [33]. KpsD is a 60 kDa outer membrane protein involved in the export of group 2 capsular polysaccharides across the outer membrane [34]. Mutation of *kpsD* results in the loss of surface expression of the capsule and accumulation of polysaccharide within the periplasmic space [35].

Given the importance of O antigen and capsule in UPEC virulence, we studied the function of these polysaccharides in three well-characterized UPEC strains (CFT073, RS218 and 1177) bearing different O and K antigens. We employed a recently described counter-selection strategy based on streptomycin-kanamycin resistance (mediated by the *rpsL* and *neo* genes, respectively) for the mutation of single genes and the subsequent repair of these mutations to generate complemented strains [36]. This enabled us to generate a defined set of wild-type (WT), mutant and complemented strains that were used to test the contributions of O antigen and capsule to UPEC virulence. Our study is the first to demonstrate the use of the *rpsL-neo* counter-selection system in UPEC, and shows that the O antigen is the major polysaccharide responsible for UPEC serum resistance and survival in the mouse urinary tract.

## Results

### Construction of capsule and O antigen insertion mutants

**Figure 1:**
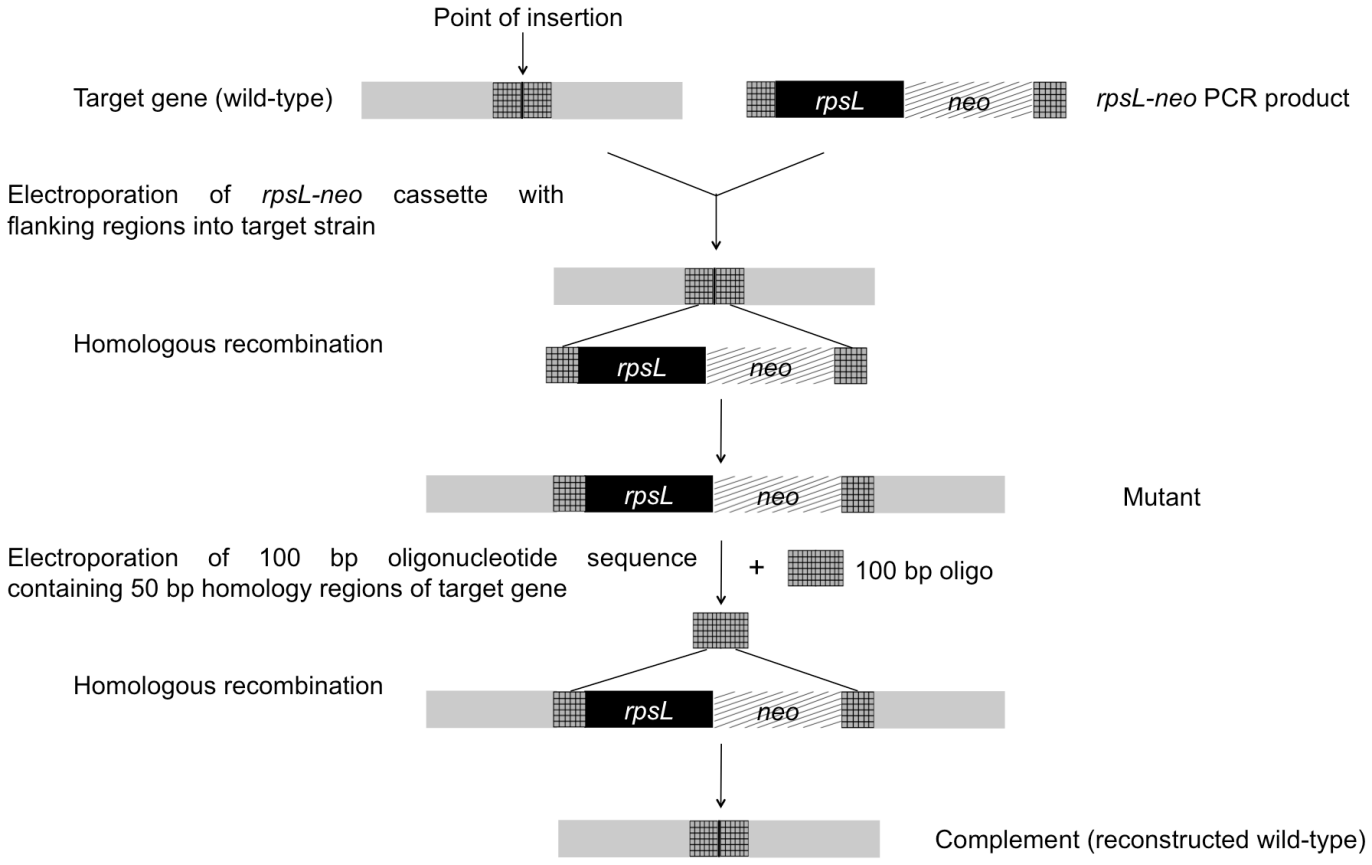
Schematic representation of the mutagenesis strategy utilizing *rpsL* counter selection. A 100(shown as patterned region in WT sequence) was selected for the *kpsD* and *waaL*; Strep^R^ strains harboring the pRedET plasmid were transformed with PCR product consisting of the *rpsL-neo* cassette flanked by 50 bp regions homologous to sequences on either side of the chosen point of insertion; double cross-over events during homologous recombination make the mutant strains Strep^S^ Kan^R^ due to integration of the *rpsL-neo* cassette; for complementation, mutant strains were transformed with 100 bp oligonucleotides bearing the same sequence as the WT across the region of insertion; complemented strains were again Strep^R^ due to loss of the *rpsL-neo* cassette and restoration of the parental genotype; streptomycin resistant (Strep^R^), streptomycin sensitive (Strep^S^), kanamycin resistant (Kan^R^). The methodology depicted in this figure is a modification of the *rpsL* counter-selection technique previously used for site-directed mutagenesis in bacterial artificial chromosomes [57]–[59].

A prerequisite for the *rpsL-neo* counter-selection system is that the bacterial strain should be streptomycin resistant (Strep^R^) and that this resistance is the direct effect of a mutation within the *rpsL* gene. Streptomycin resistance is a recessive trait and in the presence of both WT and mutant alleles, the strain is sensitive to streptomycin (Strep^S^) [37]. This trait can be exploited for the rapid mutation and repair of mutated genes, and a schematic outline of the strategy we employed is presented in Figure 1. Strep^R^ derivatives of the UPEC strains CFT073 (O6:K2), 1177 (O1:K1) and RS218 (O18:K1) were generated spontaneously by sequential plating on increasing concentrations of streptomycin. These Strep^R^ strains (CFT073^Strep^, 1177^Strep^, RS218^Strep^) were then transformed with pRedET, a temperature sensitive expression plasmid harboring the λ-Red recombinase genes. Mutation of the *waaL* (O antigen) and *kpsD* (capsule) genes in the pRedET-containing strains was performed by insertional inactivation following homologous recombination between the bacterial chromosome and the *rpsL-neo* cassette flanked by 50 bp homology regions and subsequent growth on kanamycin LB-agar. These mutants, which became sensitive to streptomycin due to the presence of a functional *rpsL* gene, were referred to as CFT073*waaL*, CFT073*kpsD*, 1177*waaL*, 1177*kpsD*, RS218*waaL* and RS218*kpsD*, respectively.

### Generation of complemented strains

To repair the mutation and generate complemented strains, pRedET was re-introduced into the mutant strains and they were transformed with a 100 bp oligonucleotide that contained the same sequence as the WT strain in the regions flanking the point of insertion of the *rpsL-neo* cassette (Figure 1). The re-constructed WT strains were selected based on resistance to streptomycin, as loss of a functional *rpsL* gene reverted them to a Strep^R^ phenotype. These strains were referred to as CFT073*waaL^C^*, CFT073*kpsD^C^*, 1177*waaL^C^*, 1177*kpsD^C^*, RS218*waaL^C^* and RS218*kpsD^C^*, respectively. In summary, we therefore generated a series of O antigen and capsule mutants, as well as repaired/complemented strains, in three UPEC strains with different O and K antigens. Hereon, all references to WT strains denote the Strep^R^ derivatives of CFT073, *E. coli* 1177 and RS218, respectively.

### Phenotypic characterization of capsule mutants and complements

**Figure 2:**
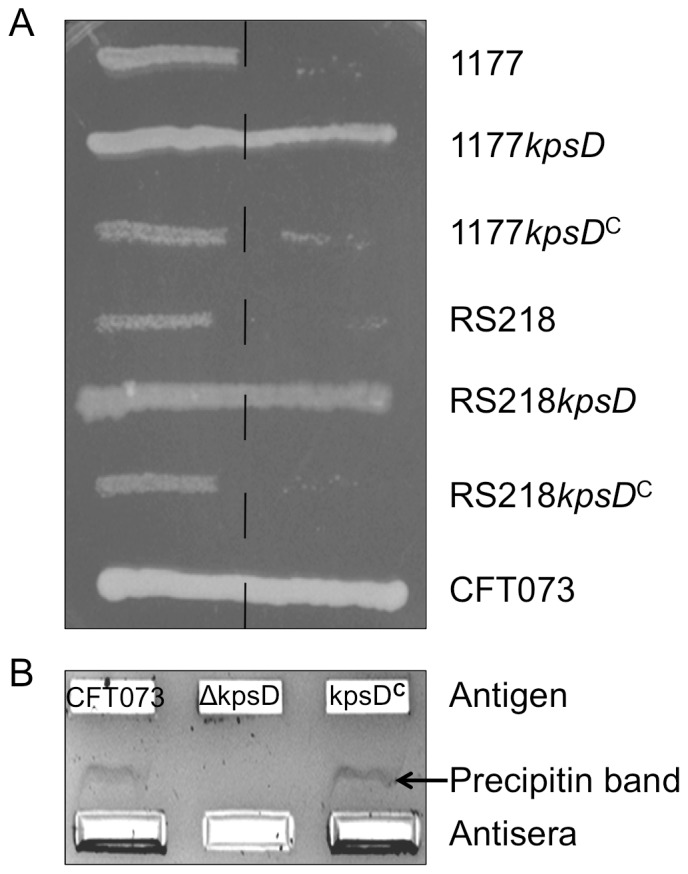
Phenotypic characterization of capsule mutants (*kpsD*) and complemented (*kpsD* ^C^) UPEC strains. A) Agar slab showing K1 phage reactions of 1177 and RS218 strains with CFT073 (expressing K2 antigen) as negative control; dashed lined through the center denotes line of phage suspension. Wild-type and complemented strains expressing K1 antigen are lysed by the phage and exhibit inhibited growth upon crossing the line of phage suspension. Growth of capsule mutants and the negative control strain is unaffected. B) Agarose gel showing precipitin bands arising from cross-linking of K2 antigen and antisera post electrophoresis. CFT073 WT and capsule complement show a positive reaction; no band is observed for the *kpsD* mutant due to loss of surface expression of the K2 antigen.

The K1 phage assay was used to detect the presence of a K1 capsule on the surface of UPEC strains 1177 and RS218 (Figure 2). In this assay, the 1177 and RS218 WT strains were killed upon encountering the phage due to its specific attachment to the K1 capsule and subsequent lytic replication life-cycle. In contrast, the *kpsD* mutants exhibited normal growth beyond the streak line, indicating a loss of K1 capsule expression and thus resistance to phage killing. A WT phenotype was fully restored in the complemented strains. In separate experiments, counter-current immunoelectrophoresis using K2 antiserum was used to detect K2 capsule on the surface of CFT073. Capsular extracts from CFT073, CFT073*kpsD* and CFT073*kpsD^C^* were electrophoresed against K2 antisera. A precipitin band was observed for CFT073 and CFT073*kpsD^C^*, while this was absent in CFT073*kpsD*. Taken together, these results confirm the K1 or K2 capsule phenotype of the three mutated and complemented UPEC strains.

### Phenotypic characterization of O antigen mutants and complemented strains

**Figure 3:**
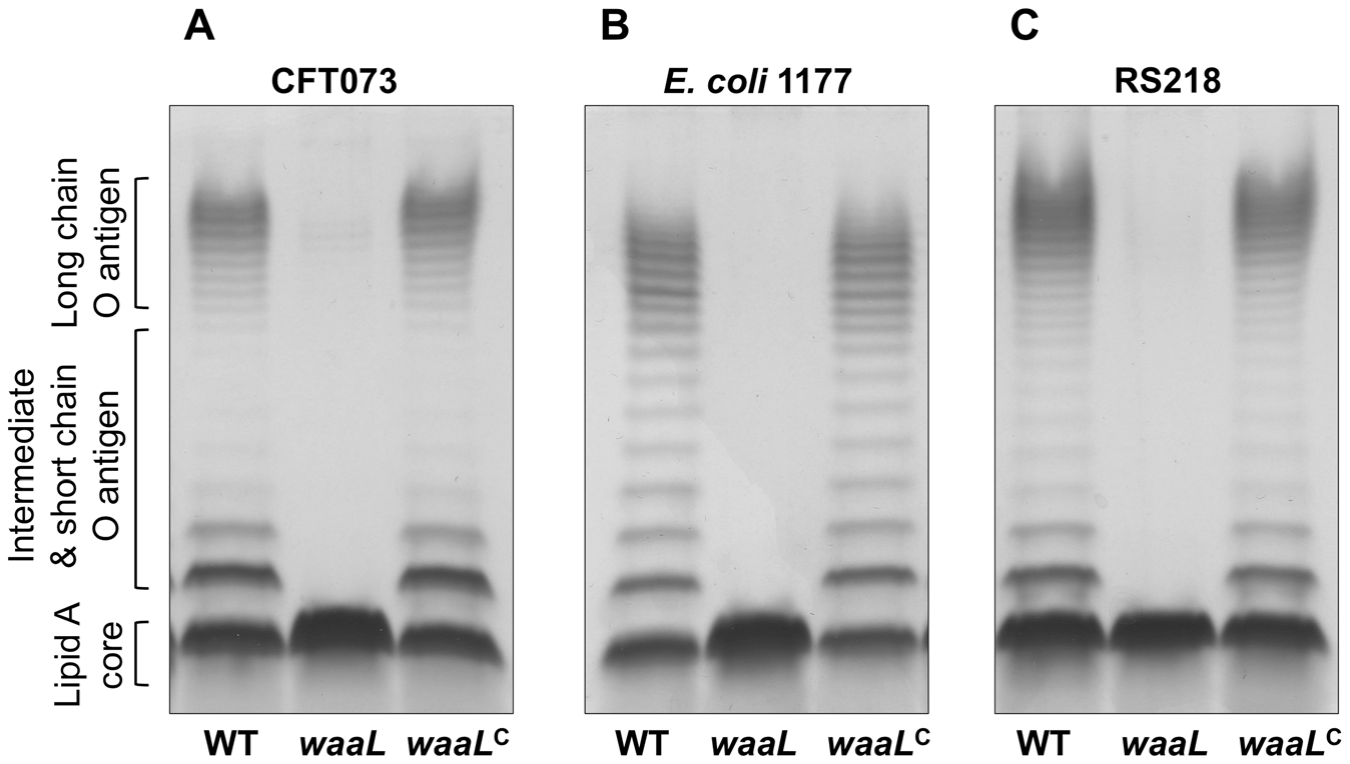
Analysis of lipopolysaccharide profiles by silver staining. Silver stained LPS profiles from strains listed below the gels. LPS banding patterns vary according to serotype; CFT073:O6 (A), *E. coli* 1177:O1 (B), RS218:O18 (C). Wild-type, O antigen mutant (*waaL*) and its complemented strain (*waaL* ^C^) are ordered from left to right. The thick band at the bottom represents the lipid A core and is intact in all the strains. The bands stacked on top of the core represent different O antigen side chains of different lengths that disappear in the *waaL* mutant strains. Banding patterns are restored in the complemented strains and appear to be the same as the WT.

The *waaL* gene encodes an O antigen ligase and its mutation abolishes the attachment of the O antigen to the LPS core, resulting in a rough phenotype. In order to phenotypically characterize the *waaL* mutants and their complemented strains, LPS extracts were prepared from standardized late log phase cultures, separated by TSDS-PAGE and viewed by silver staining (Figure 3). As expected, due to their different O types, the LPS banding patterns from WT CFT073, 1177 and RS218 varied. *E. coli* 1177 (O1) possessed an even distribution of short, medium and long O antigen side chains, while CFT073 (O6) and RS218 (O18) possessed predominantly short and long O antigen side chains. Disruption of the *waaL* gene resulted in complete loss of the bands corresponding to the O antigen subunits from all strains, with only the lipid A-core detected. In each of the complemented strains, the O antigen-banding profile was restored to WT.

### O antigen is the major determinant of serum resistance

**Figure 4:**
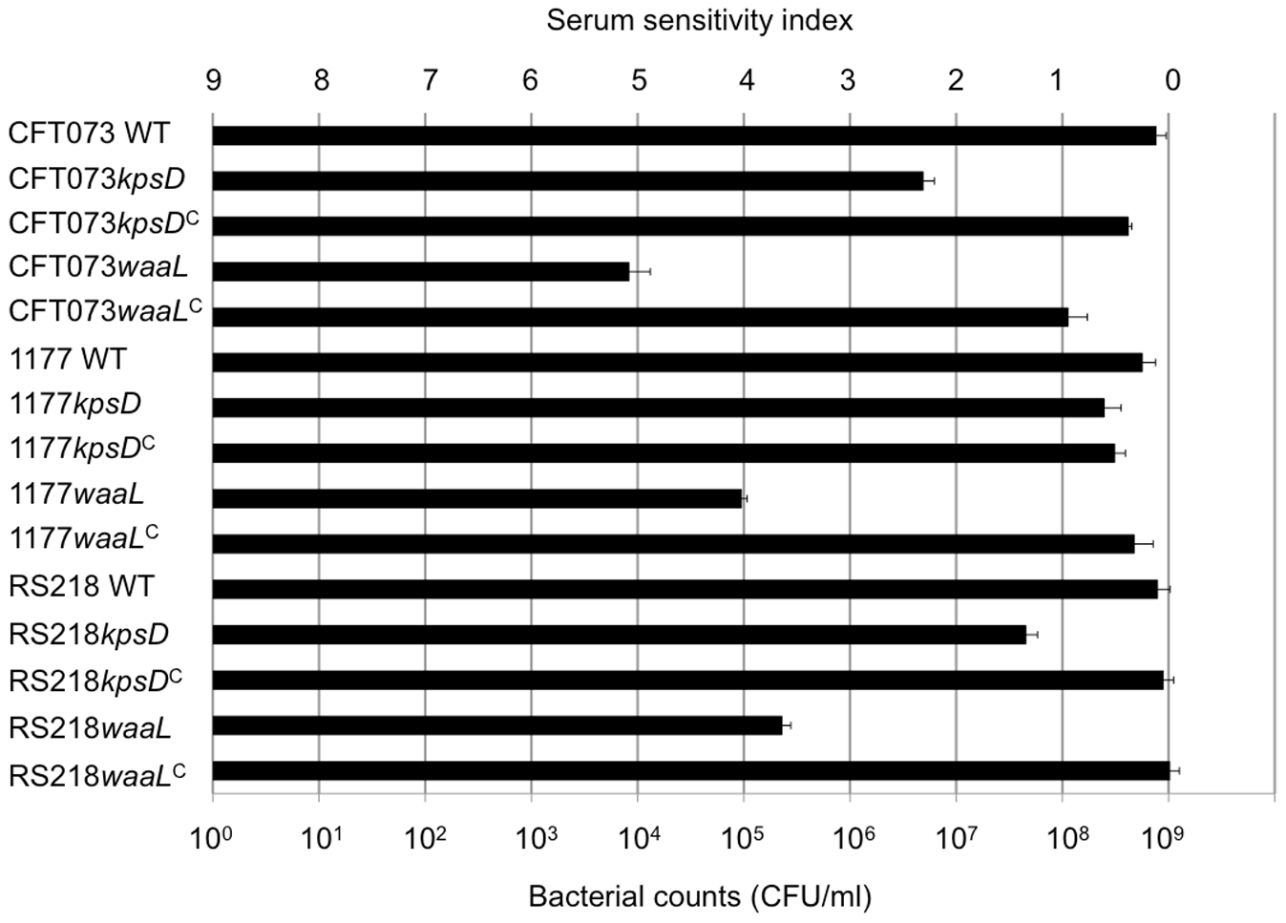
UPEC survival in human serum. Bacteria (approximately 4×10^8^ CFU/ml) were incubated in fresh human serum for 90 min and colony counts were used to determine serum sensitivity index (SSI) for each strain, shown at the top of the graph. SSI of 0 represents a resistant strain; 1,2,3,4,5 represent equivalent reduction in log values of colony forming units (CFU/ml) post incubation with serum. The WT strains CFT073, 1177 and RS218 were resistant; CFT073*waaL*, 1177*waaL* and RS218*waaL* mutants were all highly sensitive to human serum (SSI; *P*<0.05, paired t test) and the complemented strains (*waaL* ^C^) were resistant. CFT073*kpsD* and RS218*kpsD* mutants displayed reduced sensitivity (SSI = 2 and 1, respectively); complemented strains (*kpsD* ^C^) were resistant. The 1177*kpsD* mutant was completely resistant to serum killing.

To compare the relative contribution of O antigen and capsule to UPEC serum resistance we tested our WT, mutant and complemented strains in a serum survival assay. In this assay, bacterial survival was measured as colony forming units (CFU) before and after incubation with pooled normal human sera. As expected, the WT strains CFT073, 1177 and RS218 were resistant to killing by human serum (Figure 4). In contrast, the CFT073*waaL*, 1177*waaL* and RS218*waaL* mutants were all highly sensitive to human serum and displayed a high serum sensitivity index (SSI; *P*<0.05, paired *t* test). This phenotype reverted back to WT (i.e. resistant) in each of the respective complemented strains. The contribution of the capsule to serum resistance was less clear. The CFT073*kpsD* mutant was most sensitive (SSI = 2, *P* = 0.046), while RS218*kpsD* displayed reduced sensitivity (SSI = 1, *P* = 0.027). Complementation of the *kpsD* mutation in CFT073*kpsD* and RS218*kpsD* resulted in reversion to a WT resistance phenotype. The 1177*kpsD* mutant, on the other hand, was completely resistant to serum killing. The *E. coli* K12 strain MG1655 was used as a control for the integrity of serum, and was completely killed in all assays. All the strains were resistant to heat-inactivated normal human sera (data not shown). Taken together, our data show that O antigen is the major determinant of serum resistance in the UPEC strains tested. Furthermore, given that 1177 and RS218 are K1 capsule type, the observed difference in their serum sensitivities indicate that the contribution of the capsule to serum resistance is closely linked to O antigen serotype.

### UPEC survival in whole blood

**Figure 5:**
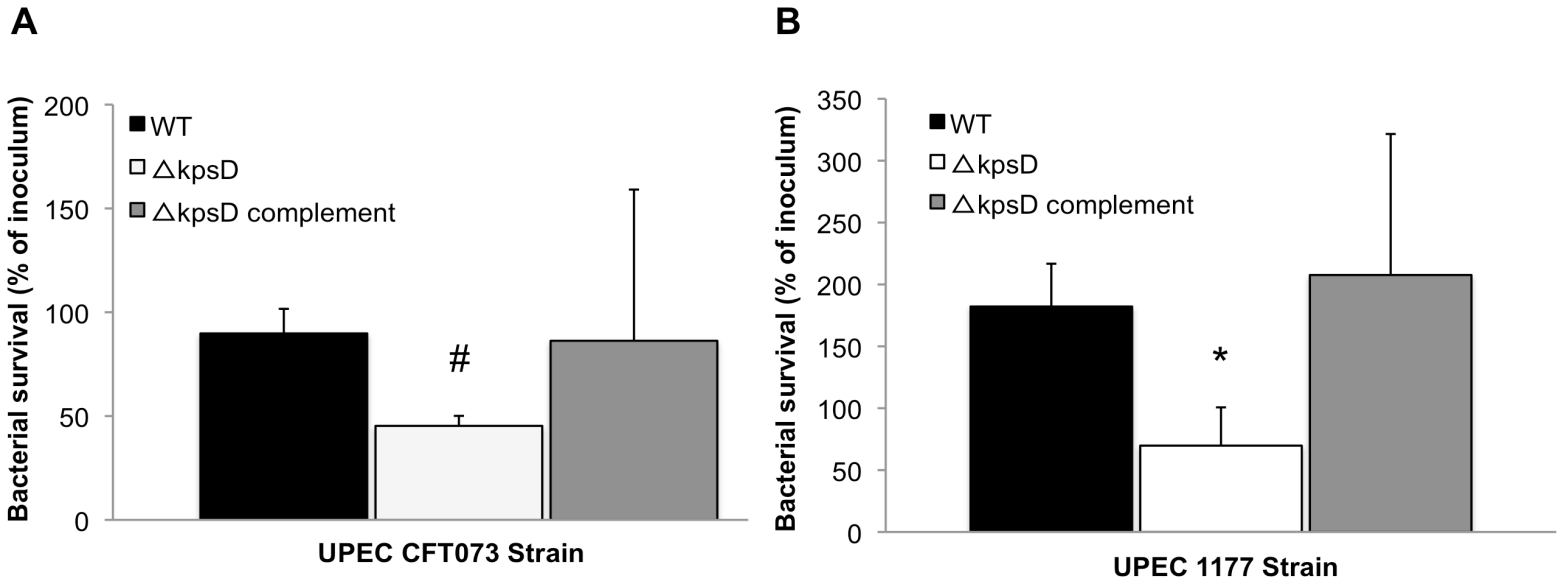
Survival of UPEC CFT073 (A) and 1177 (B) WT, mutant and complemented strains in whole blood killing assays. Bacteria were incubated in fresh whole blood for 3–5 h and colony counts were used to compare survival phenotypes of mutants and complemented strains to the WT parents. Fitness of the CFT073 and 1177 capsule (*kpsD*) mutants was significantly impaired compared to the WT, and was restored completely in the respective capsule complemented (*kpsD* ^C^) strains. The survival data for the RS218 WT derivatives could not be analyzed due to the high sensitivity of WT strain to killing by whole blood. *P* values, as determined by the independent samples, t test were as follows: # 0.038, * 0.042.

The most well-characterized virulence-related role of capsular expression in UPEC relates to bacterial resistance to uptake and killing by phagocytes such as neutrophils and monocytes [15]. In view of this, and the data from the above assays that showed a less clear contribution of the capsule to serum resistance compared to O antigen, we performed a series of whole blood killing assays. These assays incorporate both of the cell types relevant to UPEC phagocytosis. In these experiments, the CFT073*kpsD* and 1177*kpsD* mutants displayed significantly enhanced sensitivity to killing by whole blood compared to their respective WT counterparts (Figure 5; *P*<0.05, independent samples t test); in contrast, RS218 WT was so sensitive to killing in this assay that the data on the *kpsD* mutants could not be interpreted. Complementation of the *kpsD* mutation in CFT073*kpsD* and 1177*kpsD* restored survival to WT levels. Given that 1177 and RS218 both produce a K1 capsule, these data suggest that there may be differences in the composition of their K1 antigen, or that the role of the K1 capsule in resistance to killing by whole blood is strongly linked to O antigen type, as observed in serum killing assays.

### The O6 antigen and K2 capsule are required for colonization of the mouse urinary tract

**Figure 6:**
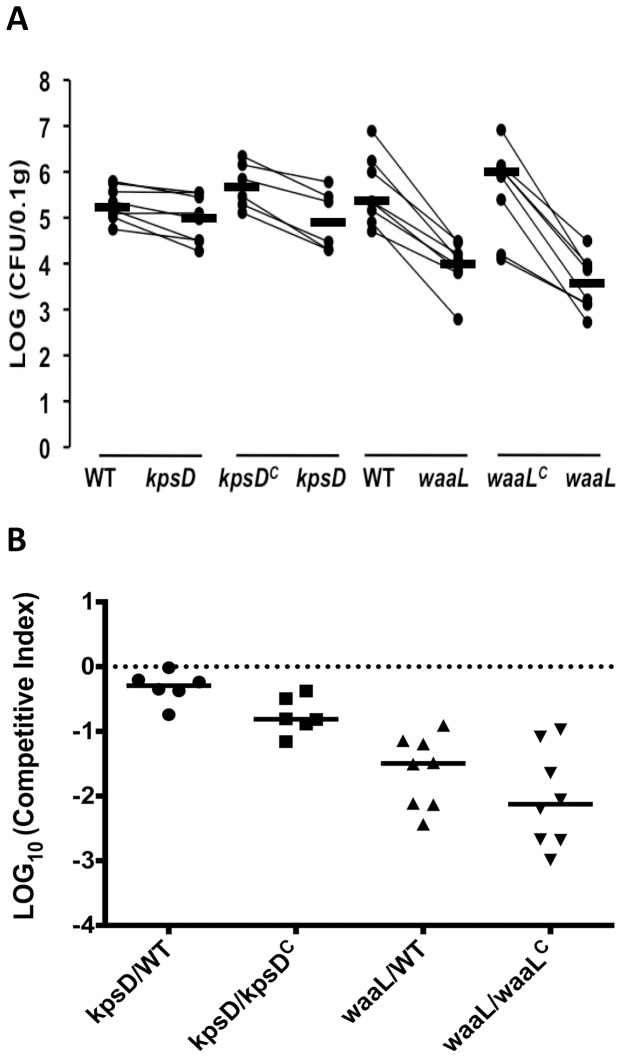
Fitness of CFT073 capsule (*kpsD*) and O antigen (*waaL*) mutants during *in vivo* competition with CFT073 WT and corresponding complemented (*kpsD* ^C^, *waaL* ^C^) strains. C57B/L6 mice were infected transurethally with a 1∶1 mixture of CFT073*kpsD* and CFT073 WT or CFT073*kpsD* ^C^; CFT073*waaL* and CFT073 WT or CFT073*waaL* ^C^. (A) Each marker represents LOG_10_ total CFU recovered from each mouse per 0.1 g of bladder tissue. Lines connect data points for the same mouse, and horizontal bars represent median values. (B) Each marker represents the LOG_10_ competitive index calculated for each individual mouse; competitive indices are the ratio of the mutant 0.1 g of bladder tissue to that of WT or complemented strain. Dashed lines represent hypothetical competitive index of 1 (LOG_10_1 = 0), which indicates no difference in fitness between the two strains. Horizontal bars represent group medians, and each competition group had 6 to 8 mice. All mutants were significantly outcompeted by CFT073 WT or their respective complemented strains for bladder colonization (*P*<0.05). The O6 antigen was significantly more important than the K2 capsular antigen for bladder colonization (*P<*0.05).

Analysis of plasmid-based complemented strains *in vivo* is challenging as it is difficult to ensure consistent expression of the cloned gene or maintenance of the plasmid in the pathogen during infection. A major advantage of the counter selection strategy described here is that complementation is performed by repair of the mutated chromosomal gene, and thus the complemented strains can be tested *in vivo*. In order to demonstrate this, we analyzed the CFT073 WT, capsule and O antigen mutant and complemented strains in a mouse UTI model. Experiments were performed as mixed competition assays, whereby mice were inoculated with a 1∶1 mix of CFT073:CFT073*kpsD,* CFT073*kpsD*:CFT073*kpsD* ^C^, CFT073:CFT073*waaL* or CFT073*waaL*:CFT073*waaL* ^C^ and the number of colonizing bacteria was calculated at 18 h post infection. In these assays, both the capsule and O antigen mutants were significantly outcompeted by the WT and complemented strains in colonization of the bladder (Figure 6; *P*<0.05). No significant colonization of the kidneys was observed (data not shown); this is consistent with previous data from our laboratory using C57BL/6 mice [38]–[41]. Taken together, these data demonstrate that the O6 antigen has a major impact on the colonization of the urinary tract by CFT073, and that the K2 capsular antigen, while less important, also contributes to this phenotype.

## Discussion

The polysaccharide capsule and O antigen are well-described UPEC virulence determinants that contribute to resistance against host defenses. Specific O and K types are associated with UPEC infection, often to different degrees depending on the serotype of the strain. In this study, we investigated the role of different O and K antigens in mediating resistance to serum and whole blood, and colonization of the urinary tract, using a novel set of WT, mutant and complemented strains.

Gene disruption in bacteria using λ-Red mediated homologous recombination is a widely used and highly efficient technique [42]. In this method, the mutation of a gene or set of genes is attained by insertional inactivation using an antibiotic cassette that can subsequently be removed by FLP-mediated recombination to leave a characteristic scar sequence at the target site. Several modifications have been made to λ-Red recombineering to improve recombination frequency that can otherwise be a major limitation in certain applications. These include using transformation amplimers with large flanking regions [43], the removal of endogenous nucleases from the host strain [44], and the use of three-way overlapping PCR methods that negate the need for multiple intermediary cloning steps [45], [46]. Complementation of a specific mutation, a common procedure used to prove the function of a gene and fulfill Koch's postulates, is normally achieved *in trans* by introduction of the relevant gene into the cell on a plasmid. This approach, while effective, can be complicated by the need for antibiotic selection to maintain the plasmid [47], [48], differences in copy number of the complemented gene due to its location on a plasmid [49] and differences in expression of the complemented gene [50]. These issues are often compounded when complementation is attempted in an animal infection model. Another approach for complementation involves the use of a transducing phage to repair the mutation on the chromosome [51]. This involves the replacement of up to 40 kb of the chromosomal region surrounding the target gene, and also requires the use of a strain-specific phage. A different allelic exchange strategy using suicide vectors can be employed when several rounds of modification are required, however problems such as the need for large flanking regions to facilitate recombination, loss of temperature sensitivity of the vector or drastically reduced recombination efficiency due to mismatched bases with target DNA sequences make this method less than ideal when dealing with non genome-sequenced clinical isolates [52]. Transposable elements also provide an alternative to λ-Red mediated homologous recombination. Transposition based strategies involving transposons such as Tn-5 and Tn-7, as well as their derivatives, have been used successfully to generate transposon mutant libraries [53], [54] for site specific tagging [55] and for the generation of transcriptional/translational target gene fusions [56].

The *rpsL* counter selection system has been used previously for the modification of bacterial artificial chromosomes in viral and eukaryotic systems [57]-[59]. In *E. coli*, this system has been used in conjunction with *loxP* to generate markerless chromosomal deletions [60] and to introduce point mutations at specific sites on the chromosome [36]. Here, we have demonstrated its application in the construction of mutant and complemented UPEC strains. The method for complementation was highly efficient, and only required the use of a 100 base oligonucleotide that matched the target region. Since the complementation method stably generates the parental genotype without the need for a selectable marker, complemented strains can be used in animal infection experiments without antibiotic selection. This method is analogous to that previously described for *sacB*, another widely used counter selectable marker [61]. However, an advantage of *rpsL* counter selection is that unlike the *sacB* system, it can still be used in strains containing a native ColE1 replicon [62], [63]. A disadvantage of the *rpsL* counter selection system is that it requires the generation of a spontaneous streptomycin resistant derivative of the strain to be studied, and it cannot be applied directly to strains with an existing streptomycin resistance cassette [64], [65]. Mutations in the native *rpsL* gene have also been shown to result in a fitness cost on *E. coli* growth, with the decline in bacterial fitness dependent on the type of *rpsL* mutation and the carbon source available in the medium [66], [67]. Thus, this issue needs to be considered when comparing the virulence of knockout and complemented strains where one is Strep^R^ and the other is Strep^S^. In this study, we used spontaneously generated Strep^R^ derivatives of each parent strain (referred to as WT for simplicity), as well as mutants and repaired/complemented strains generated from these Strep^R^ derivatives. Although we did observe a slight decrease in exponential growth rate for the Strep^R^ strains, several of our mutants (which were Strep^S^) still exhibited clear phenotypes with respect to serum resistance, whole blood killing and bladder colonization. Furthermore, our spontaneous CFT073 Strep^R^ derivate (CFT0073^Strep^) colonized the C57BL/6 mouse bladder at a similar level to that we have reported previously for CFT073 [38], [68].

Three UPEC strains with different O and K antigens were used to demonstrate the application of the *rpsL* counter selection system. For UPEC, the potential influence of *waaL* deletion and disruption of expression of O antigen on virulence is undefined except for a recent study that showed *waaL* was required for efficient colonization of mouse bladder [14]. Disruption of the *waaL* gene has been shown to result in uncapped lipid A-core due to the absence of a functional O antigen ligase [23], [69]. Our *waaL* mutants had a similar lipid A-core region, while the characteristic O antigen was absent (and restored in complemented strains). The role of O antigen in mediating serum resistance in multiple bacterial pathogens has been extensively reported in the literature [32], [43], [70], [71]. Our results demonstrate that the O1, O6 and O18 antigens are important for survival in serum. This is consistent with previous studies on the O18 antigen, which has been extensively studied in the context of neonatal meningitis strains [72]–[74]. Our results for the O1 and O6 antigens, while expected, represent to the best of our knowledge the first demonstration of this phenotype in well-defined mutant and complemented strains.

Mutation of the *kpsD* gene resulted in mutants lacking any surface exposed K antigen, and this was restored by complementation. In our experiments, the K2 capsule contributed to the resistance of CFT073 against the bactericidal activity of human serum. A study by Buckles *et al*. has shown the importance of the K2 capsule in UTI pathogenesis and its role in preventing complement mediated killing [31]. Notably, the serum sensitivity index for the K2 mutant in our study was significantly lower than its corresponding O antigen mutant (*P*<0.01). However, both of the strains were resistant to heat-inactivated normal human sera indicating a role for complement attack that depends at least in part, on the presence of O antigen. There are conflicting reports in the literature about the importance of K1 capsule in effecting bacterial survival in serum. A study by Leying *et al.* has suggested that K1 expression is a prerequisite for serum resistance [28]. However, the mutants used were not defined and complementation was not described. Likewise, Kim *et al.* reported that the K1 capsule plays a more important role than the O18 antigen in establishing bacteremia in a neonatal rat meningitis model [29]. In contrast, an earlier report concluded that in a similar animal model, the K1 capsule, while important for establishing meningitis, had no effect on bacterial survival in serum [75]. Several other reports have corroborated these findings [30], [76], [77]. Both the RS218 and 1177 strains used in our study produce a K1 capsule. We observed that while the absence of the capsule renders RS218 slightly susceptible to the bactericidal activity of serum, it had no significant effect on 1177. We also analyzed UPEC survival in whole blood to elucidate the role of capsule in protecting against whole blood killing. Survival of the CFT073 and 1177 capsule mutants was significantly impaired, which is similar to the role of K5 capsule antigen that mediates resistance to phagocytosis in UPEC [15]. The RS218 WT strain exhibited a poor survival rate and its corresponding mutants could not be analyzed by this assay. Thus, the importance of the K1 capsule in mediating serum resistance and survival in whole blood is variable, and possibly linked to the O antigen type and other factors such as hemolysin [78], outer membrane proteins (OmpA, OmpC) [79], [80] and plasmid-encoded virulence factors (TraT, Iss) [81], [82].

A key advantage of the *rpsL* counter selection strategy is that mutants can be complemented and studied *in vivo*. To demonstrate this, the ability of the CFT073 capsule and O antigen mutant strains to cause disease in a mouse UTI model was analyzed. We observed that both the K2 and O6 mutants were outcompeted by the WT and the complemented strains in bladder colonization during mixed infection challenges. However, based on competitive index the O6 antigen was significantly more important for disease pathogenesis than the K2 capsule. These results confirm the importance of the O6 antigen and, to some extent, the K2 capsular antigen, as colonization factors of the mouse urinary tract. The results are also consistent with a recent report demonstrating that restoration of the O-antigen producing capacity of the *E. coli* K12 strain MG1655 was sufficient for it to kill *Caenorhabditis elegans* at rates comparable to other pathogenic bacteria [83]. The expression of O antigen and capsule on the bacterial surface can also affect the function of other virulence factors [84], [85]. Differences in the role of other UPEC K capsular antigens during infection have also been described previously. For example, the K15 antigen is important for virulence but not for serum resistance in a neonatal mouse UTI model [86], while a protective role for O4 but not for the K54 antigen has been described during colonization of the mouse urinary tract [87].

The O antigen and capsule are well known virulence factors of UPEC, however the interplay between common O and K antigens remains to be assessed using defined mutant and complemented strains. Here we have described a strategy to make single gene mutants and their complements that can be analyzed *in vitro* and *in vivo*. With the aid of mutant and complemented strain sets we have shown that the surface expression of the O1, O6 and O18 antigens and to a lesser extent, the K2 antigen, are important for bacterial survival in human serum. O1, O6, K1 and K2 antigens were all found to provide a survival advantage in the presence of whole blood. The O6 antigen was shown to be more important than the K2 antigen for colonization of the mouse bladder. The strategy described herein can be applied to study other virulence factors with complex functions and thus avoid the use of plasmid-based systems for complementation.

## Materials and Methods

### Ethical approval

This study was carried out in strict accordance with the recommendations in the Animal Care and Protection Act (Queensland, 2002) and the Australian Code of Practice for the Care and Use of Animals for Scientific Purposes (7th edition, 2004). Approval for mouse infection studies was obtained from the University of Queensland Animal Ethics Committee (SCMB/471/09/NHMRC (NF)). Approvals for the collection of human blood for serum sensitivity and whole blood killing assays were obtained from the University of Queensland Medical Research Ethics Committee (2008001123) and the Griffith University Human Research Ethics Committee (MSC/18/10/HREC), respectively.

### Bacterial strains and growth conditions

The UPEC strains used in this study are listed in Table 1. Wild-type clinical isolates and their derivatives were routinely cultured at 28°C or 37°C on solid or in liquid Luria-Bertani (LB) medium [88] supplemented with the appropriate antibiotics: streptomycin (Strep, 100 μg/ml), kanamycin (Kan, 50 μg/ml), tetracycline (Tet, 5 μg/ml). Streptomycin-resistant derivatives of UPEC strains CFT073, 1177 and RS218 were generated by sequential growth on increasing concentrations of streptomycin (10 μg/ml, 25 μg/ml, 50 μg/ml and 100 μg/ml).

**Table 1:** Bacterial strains used in this study

Strain	Relevant Characteristics	Reference
***E. coli*** ** K12 strains**		
MG1655	OR:K-	[94]
MC1061*hsdR*	*hsdR, mcrB, araD,* 139_(araABCleu) 7679 ΔlacX74 galU galK rpsL thi	[47], [95]
**UPEC WT strains**		
CFT073	O6:K2	[96]
*E. coli* 1177	O1:K1	[97]
RS218	O18:K1	[98]
**Streptomycin resistant strains**		
CFT073 Strep^R^	CFT073*rpsL*, Strep^R^	This study
*E. coli* 1177 Strep^R^	*E.coli* 1177*rpsL*, Strep^R^	This study
RS218 Strep^R^	RS218*rpsL*, Strep^R^	This study
**CFT073 strains**		
CFT073(pRedET)	CFT073*rpsL* (pRedET), Strep^R^, Tet^R^	This study
CFT073*kpsD*	CFT073*kpsD*::*rpsl-neo*, Kan^R^	This study
CFT073*kpsD*(pRedET)	CFT073*kpsD*::*rpsl-neo*(pRedET), Kan^R^, Tet^R^	This study
CFT073*kpsD* ^C^	Re-constructed CFT073 from CFT073*kpsD*, Strep^R^	This study
CFT073*waaL*	CFT073*waaL*::*rpsl-neo*, Kan^R^	This study
CFT073*waaL*(pRedET)	CFT073*waaL*::*rpsl-neo*(pRedET), Kan^R^, Tet^R^	This study
CFT073*waaL* ^C^	Re-constructed CFT073 from CFT073*waaL*, Strep^R^	This study
*E. coli* 1177 strains		
1177(pRedET)	1177*rpsL* (pRedET), Strep^R^, Tet^R^	This study
1177*kpsD*	1177*kpsD*::*rpsl-neo*, Kan^R^	This study
1177*kpsD*(pRedET)	1177*kpsD*::*rpsl-neo*(pRedET), Kan^R^, Tet^R^	This study
1177*kpsD* ^C^	Re-constructed 1177 from 1177*kpsD*, Strep^R^	This study
1177*waaL*	1177*waaL*::*rpsl-neo*, Kan^R^	This study
1177*waaL*(pRedET)	1177*waaL*::*rpsl-neo*(pRedET), Kan^R^, Tet^R^	This study
1177*waaL* ^C^	Re-constructed 1177 from 1177*waaL*, Strep^R^	This study
RS218 strains		
RS218(pRedET)	RS218*rpsL* (pRedET), Strep^R^, Tet^R^	This study
RS218*kpsD*	RS218*kpsD*::*rpsl-neo*, Kan^R^	This study
RS218*kpsD*(pRedET)	RS218*kpsD*::*rpsl-neo*(pRedET), Kan^R^, Tet^R^	This study
RS218*kpsD* ^C^	Re-constructed RS218 from RS218*kpsD*, Strep^R^	This study
RS218*waaL*	RS218*waaL*::*rpsl-neo*, Kan^R^	This study
RS218*waaL*(pRedET)	RS218*waaL*::*rpsl-neo*(pRedET), Kan^R^, Tet^R^	This study
RS218*waaL* ^C^	Re-constructed RS218 from RS218*waaL*, Strep^R^	This study

### DNA manipulations and genetic techniques

Oligonucleotides used in this study were sourced from Sigma-Aldrich (Australia). Plasmid pRedET and the *rpsL-neo* template were obtained from the Counter selection BAC modification kit (GeneBridges). Isolation of plasmid DNA was carried out using the QIAprep Spin Miniprep Kit (Qiagen). All polymerase chain reactions were performed using *Taq* DNA polymerase (New England Biolabs) under standard conditions except for reactions requiring high fidelity, in which case Phusion DNA polymerase (Thermo Scientific) was used. DNA sequencing for mutant and complement validation was performed using the Big Dye v3.1 kit (Applied Biosystems) at the Australian Equine Genetics Research Centre, University of Queensland.

### Construction of capsule and O antigen insertion mutants

Primers were designed using Vector NTI Advance 10 software (Invitrogen). Due to the unavailability of the RS218 and *E. coli* 1177 genome sequence, sequences of *kpsD* and *waaL* genes from UPEC strains CFT073, APEC01, 536 and UTI89 were aligned to identify 100 bp regions of complete sequence conservation. These sequences were incorporated as the 50 bp homology regions at the 5′ end of the forward and reverse knockout primers. The 3′ end of the primers contained the 24 base sequences specified in the Counter selection BAC modification kit (GeneBridges), which were used to amplify the *rpsL-neo* cassette. The primer sequences used are as follows: *kpsD* forward (F): TCCAACGTCAACGTCTACGCCTCCTTATTACAGGCGCAGCCAGTAAAAGTGGCCTGGTGATGATGGCGGGATCG; *kpsD* reverse (R): ACGTCACACCGCCATACAGACCAGGATTACGCACAAATCCGGTCACGTACTCAGAAGAACTCGTCAAGAAGGCG; *waaL* F:

CGCGTGCAGCCATATTAGTGTTTCCATTCTTTGCGTTACTATTAATCGTAGGCCTGGTGATGATGGCGGGATCG; *waaL* R: ATAAAACAATATAACTTATAATTAATTCGCTTATTAATATAACTATCCATTCAGAAGAACTCGTCAAGAAGGCG. Screening primers were designed at least 70 bp upstream and downstream of the homology regions, respectively. Their sequences are as follows: *kpsD* screen F: GATGGTGCGTTACAGGTTGATC; *kpsD* screen R: CCGCGCTTGACCACAATATC; *waaL* screen F: CAACAGCAGGCTACATCATCC; *waaL* screen R: CTTCATACATTGCCAGACGG. Streptomycin resistant UPEC strains CFT073, 1177 and RS218 were transformed with pRedET by electroporation and selected in the presence of tetracycline. These strains were then grown at 28°C to an optical density at 600nm (OD_600 nm_) of 0.3, induced with L-arabinose (0.3% w/v) for approximately 1 h to allow expression of the recombinase genes, and then electroporated with the *rpsL*-*neo* cassette bearing the 50 bp homology regions. Mutants that were streptomycin sensitive (Strep^S^) and kanamycin resistant (Kan^R^) were identified and insertion of the *rpsL-neo* cassette within the *kpsD* and *waaL* genes, respectively, was confirmed by PCR and sequencing.

### Complementation of capsule and O antigen insertion mutants

Plasmid pRedET was re-introduced into the CFT073, 1177, and RS218 *waaL* and *kpsD* mutants, respectively. To repair the *waaL* and *kpsD* mutations, these strains were prepared as described above and transformed with 2 μM single-stranded 100 base oligonucleotides comprising a sequence identical to the WT strain in the mutated region (*kpsD*:TCCAACGTCAACGTCTACGCCTCCTTATTACAGGCGCAGCCAGTAAAAGTGTACGTGACCGGATTTGTGCGTAATCCTGGTCTGTATGGCGGTGTGACGT; *waaL*:CGCGTGCAGCCATATTAGTGTTTCCATTCTTTGCGTTACTATTAATCGTAATGGATAGTTATATTAATAAGCGAATTAATTATAAGTTATATTGTTTTAT). Repair of the specific *waaL* or *kpsD* mutation in Strep^R^ colonies was confirmed by PCR screening and sequencing.

### K1 phage assay

K1 phage solution was sourced from the Statens Serum Institut (Denmark). The cross-brush method recommended by the manufacturer was used to determine the presence of K1 antigen [89]. Briefly, a vertical line of phage suspension was streaked across an agar plate and allowed to dry. Horizontal lines of overnight *E. coli* broth cultures were then streaked from left to right across the phage suspension and plates were incubated at 37°C for 12 h. Inhibited growth or complete killing of bacteria after crossing the phage suspension was deemed a positive reaction. A negative reaction, in the absence of K1, was characterized by normal bacterial growth across the entire bacterial streak. CFT073 WT strain with K2 capsule was used as a negative control for the assay.

### Counter current immunoelectrophoresis

*E. coli* diagnostic K2 antisera (Statens Serum Institut, Denmark) was used to detect the presence of the K2 antigen. Capsule extracts were made according to manufacturer guidelines. Briefly, the bacterial cells from an agar plate were suspended in 1 ml phosphate buffered saline (PBS) (pH 7.4) and incubated at 60°C for 20 min. The cell suspension was then centrifuged for 30 min at 14,000 rpm and the supernatant transferred to a fresh tube and heated for 1 h at 95°C. Extracts were then diluted in PBS (1∶50) for electrophoresis. A 1.5% agarose gel was applied to a glass plate with 2 rows of wells and a distance of 5 mm between each row. 15 μl of K2 antisera and diluted capsule extracts were loaded at the anode and cathode ends, respectively. Counter-current immunoelectrophoresis was performed for 70 min at 80 V in 1X TAE buffer. A precipitin band between the wells demonstrated the presence of a K2 capsule.

### LPS isolation and silver staining

LPS was extracted as described previously by West *et al*. [90]. Tricine-SDS PAGE (TSDS PAGE) was used to obtain a high resolution of LPS. Gels were cast with a 16.5% resolving phase containing 4 M urea, overlaid with a 4% stacking phase. A two-buffer system was used for electrophoresis as previously described [91]. Electrophoresis was performed in the Mini-PROTEAN gel apparatus (BioRad) at 50 V for 30 min and then at 150 V for 70 min. A modified silver staining method originally described by Tsai and Frasch [92] was used to view LPS bands. Gels were transferred to a glass tray and fixed overnight (40% ethanol, 5% glacial acetic acid) at room temperature. The fixing solution was replaced with an oxidizing solution (40% ethanol, 5% glacial acetic acid, 0.7% periodic acid) and incubated for 7 min. Gels were washed repeatedly in copious amounts of milliQ water and then stained (20 mM sodium hydroxide, 0.4% ammonium hydroxide, 0.7% silver nitrate) for 10 min. The staining solution was removed and gels were incubated in the dark in a developing solution (0.02% formaldehyde, 0.26 mM citric acid) until distinct bands were visible. Gels were then washed in water to stop the reaction.

### Serum killing assay

Overnight bacterial cultures were washed in PBS and then standardized to a final OD_600_ = 0.8 (approximately 4×10^8^ CFU/ml). A volume of 50 μl of each standardized culture was mixed with an equal volume of PBS and serially diluted for colony counts on agar to enumerate CFU prior to serum treatment (t = 0 min). Fresh blood was collected in EDTA-free vacutainer tubes on the day of the assay from two healthy human donors who gave informed consent. The same donors were used for all assays in order to reduce serum variability. Blood was allowed to clot for 15 min and the tubes were centrifuged at 1500 *g* for 10 min. A volume of 50 μl of each standardized culture (approximately 3×10^7^ CFU) was mixed with an equal volume of pooled human sera, mixed and incubated for 90 min at 37°C. All experiments were performed in triplicate. Colony counts were used to enumerate bacterial cells post treatment (t = 90 min). *E. coli* MG1655 was used as a serum sensitive control strain. All the strains were tested similarly in a parallel assay using heat-inactivated normal human sera (Millipore). Strains showing at least a log_10_ difference (i.e. 10-fold) in CFU/ml were deemed to have altered sensitivity to serum as compared to WT. Serum sensitivity indices (SSI) were calculated according to these differences. Paired samples t tests were performed on CFU/ml values at the two time points (0 min and 90 min) to determine statistical significance.

### Whole blood killing assay

Approximately 10^4^ colony forming units (CFU) of UPEC prepared from a late exponential phase culture in LB were added to 1.5 ml of whole blood collected from a healthy human volunteer using plasma preparation tubes (PPT) containing 9.0 mg K2EDTA (BD Vacutainer, NJ, USA). The infected cultures were incubated rolling at 37°C and after 3–5 h diluted blood samples were plated in triplicate onto agar to enumerate the surviving bacteria. The survival rate was calculated as a percentage of the starting inoculum ([CFU (at a given time point)/CFU (at the start)]×100) and *P* values were determined using the independent samples t test.

### Mouse model of UTI

The mouse model of UTI described by Roos *et al.* (2006) was used in this study [93]. Female C57BL/6 mice (8–10 weeks) were purchased from the University of Queensland Animal Facility and housed in sterile cages with *ad libitum* access to sterile water. All strains used in this experiment were enriched for type 1 fimbriae expression after 3 passages under static conditions. Bacterial cultures were prepared by overnight static growth in LB broth; all strains were strongly positive for type 1 fimbriae expression as determined by yeast cell agglutination. For competitive (mixed infection) assays, inocula contained 1∶1 strain mixtures of CFT073*kpsD* and CFT073 WT (group 1) or CFT073*kpsD* ^C^ (complement; group 2); CFT073*waaL* and CFT073 WT (group 3) or CFT073*waaL* ^C^ (group 4). An inoculum of 20–40 μl, containing 5×10^8^ CFU of bacteria in PBS was injected directly into the bladder of each mouse. Urine was collected from mice 18 h after challenge following which they were euthanized by cervical dislocation. Bladders were then excised aseptically, weighed, and homogenized in PBS. Urine and bladder homogenates were serially diluted in PBS and plated onto streptomycin and kanamycin-containing LB agar for colony counts. The two strains in each group were differentiated by their resistance to streptomycin (WT and complements) or kanamycin (*kpsD* and *waaL* mutants). Significance was determined using the two-tailed Wilcoxon matched pairs test on CFU/ml urine, or CFU/0.1 g bladder tissue using Prism6 (GraphPad). *P* values <0.05 were considered significant.
